# What do we mean by urgent care? Definitional confusion and its consequences

**DOI:** 10.3399/BJGP.2025.0716

**Published:** 2026-05-06

**Authors:** Anna Podlasek, Tim Sanders, Rebecca Elizabeth Payne

**Affiliations:** 1 Image Guided Therapy and Research Facility (IGTRF), University of Dundee, Dundee, UK; 2 School of Medicine, University of St Andrews, St Andrews, UK; 3 School of Medicine and Dentistry, University of Lancashire, Preston, UK; 4 North Wales Centre for Primary Care Research, North Wales Medical School, Bangor University, Bangor, UK; 5 Nuffield Department of Primary Care Health Sciences, University of Oxford, Oxford, UK

## Introduction

Few terms in contemporary NHS policy are as widely used — and as poorly defined — as *urgent care*. The phrase appears across strategic plans, service standards, and funding frameworks, yet what counts as ‘urgent’ differs markedly between organisations and settings. In England, the NHS Long Term Plan promotes ‘urgent and emergency care transformation’ as a core reform area; in Scotland and Wales, ‘unscheduled care’ or ‘urgent primary care’ frameworks fulfil similar functions.^1–4^

This lack of definitional clarity is not merely semantic. Ambiguous use of *urgent care* blurs clinical thresholds, complicates data interpretation, and undermines workforce planning. Patients face growing difficulty navigating a fragmented system where entry points, digital interfaces, and access thresholds vary widely, creating uncertainty about where, how, and when to seek help.^5^ Box 1 provides real-world examples of these challenges. These inconsistencies are not confined to policy. In research, too, the lack of a clear definition undermines comparability.^6^ UK primary care studies repeatedly link access and triage arrangements, and service configurations within general practices with urgent and emergency department (ED) utilisation by their patients.^7–9^

**Box 1.** Real-world examples of why urgent care matters

**A GP’s perspective** I work as a GP in an English UTC in a hospital that doesn’t have an ED. We are only supposed to see ‘urgent’ problems — the sorts of things that patients would otherwise see their own GP for, alongside minor injuries. However, most weeks, a patient turns up with chest pain. We don’t have the facilities to diagnose and treat myocardial infarction, so the patients must wait in our department for an ambulance, taking up staff time and delaying the care of other patients. I find this extremely stressful as some of these patients can be really sick and I worry that they will arrest or that we will be left waiting long periods for an ambulance to arrive. **A patient’s perspective** My mother fell while on holiday in North Wales. We took her to the minor injuries unit, but they couldn’t X-ray her arm, so we had to travel an hour and a half in the other direction to a busy ED. I don’t understand why they couldn’t offer this service; at home our local UTC would routinely do this. If we’d known how limited local services were, we could have saved ourselves a journey in the wrong direction by going straight to the big hospital; however, at home, that would have been the wrong thing to do. **An advanced practitioner’s perspective:** I work as an ANP in a busy English UTC and do agency shifts for other providers, including in GPOOH services. I was asked by my agency to cover a weekend on a Scottish island. When I arrived, I was shocked to find that I was the only clinician on duty and there was no GP working alongside me. I don’t see children under 3 years of age or mental health patients, but there was no one else to see them. I felt like I had been put in an impossible situation.

*ANP = advanced nurse practitioner. ED = emergency department. GPOOH = general practice out of hours. UTC = urgent treatment centre.*

This article traces the origins and evolution of the ‘urgent care’ concept in the UK, compares definitions across devolved administrations and professional bodies, and considers implications for patients, health services research, and policy.

Box 1 presents fictionalised composite vignettes developed by the authors, drawing on recurrent scenarios encountered in clinical practice and professional discussions. These are not verbatim accounts, do not represent any single individual or organisation, and are included for illustrative purposes only to demonstrate how definitional ambiguity in urgent care manifests in real-world settings.

### Origins and evolution of ‘urgent care‘ in UK policy

During the mid- to late 1990s, GPs began to struggle to sustain 24-hour care through traditional on-call arrangements within their own practices. This challenge prompted increasing collaboration and cross-cover between neighbouring practices, which in turn evolved in many areas into GP out-of-hours cooperatives. The new General Medical Services Contract, agreed in 2003 and implemented in 2004, removed the requirement for GPs to provide out-of-hours care directly, transferring responsibility to primary care trusts and intermediate services such as walk-in centres and minor injury units.^10^

These structural changes created the foundations for a more integrated urgent-care architecture, positioned between the ‘sharp end’ of general practice and the ‘blunt end’ of the emergency department. The *NHS Plan* ^11^ envisaged a single integrated urgent-care system to manage problems not requiring the emergency department but too urgent for routine general practice.^11^ By 2013, the *Urgent and Emergency Care Review* (Keogh Review) reaffirmed the ambition for a 24/7 urgent-care system in which patients know where to go and get the right care, first time.^12^ Yet neither policy articulated a formal definition of *urgent care*. Instead, the term gradually evolved into an umbrella descriptor encompassing a wide spectrum of provision — from NHS 111 and GP out-of-hours services to walk-in centres, urgent-treatment centres, and same-day access within general practice.

### Current definitions: a patchwork of policy and practice

Box 2 summarises definitions of urgent care across the four UK nations. Common elements include time-sensitive, non-life-threatening conditions and the need to prevent deterioration through timely assessment or treatment. In addition, significant confusion for patients and clinicians is likely to arise from variation within nations. Even within the same region, differing commissioning decisions, service configurations, and workforce models can result in markedly different urgent-care pathways, further contributing to the challenges navigating a fragmented system.

**Box 2.** Contemporary definitions of urgent care

Nation	Source	Definition
England	NHS England^20^	*‘Urgent care involves any non-life-threatening illness or injury needing urgent attention which might be dealt with by phone consultation through the NHS111 Clinical Assessment Service, pharmacy advice, out-of-hours GP appointments, and/or referral to an urgent treatment centre (UTC).’*
Wales	NHS Wales^21^	*‘Health and wellbeing issues that may result in significant or permanent harm if not dealt within the next 8 hours. Urgent primary care services include a phone consultation through same day/out-of-hours primary care appointments, the NHS111 Wales (including the 111 Clinical Support Hub), pharmacy advice, and/or referral to an urgent primary care centre (UPCC).‘*
Scotland	Scottish Government^4^	*‘Urgent care refers to the need for medical treatment for a condition or injury which is not considered to be imminently life threatening but could worsen if left untreated and unscheduled care describes the need for unplanned medical care often as a result of an accident.’*
Northern Ireland (NI)	NI Department of Health^22^	*’An illness or injury that requires urgent attention but is not a life-threatening situation* ***’***

Each nation currently provides at least a partial concept or definition of urgent care. Examining these definitions highlights both shared principles and potential omissions. For example, the temporal threshold for what constitutes urgent varies: from same day in England to within 8 hours in Wales, and remaining undefined in Scotland and Northern Ireland. Similarly, the locus of the perception of urgency also varies, shifting between the patient, the triaging clinician, and the capacity of local services. While all of these elements are relevant, no single definition is sufficiently comprehensive or unifying.

This highlights the limitations of local definitions and supports the need for the common language of a shared conceptual framework.

Academic literature mirrors this variability. A literature review commissioned by the Welsh Government’s Strategic Programme for Primary Care^1^ screened ~60 000 records, included 170 papers, and found only 40 with definitions or conceptual content directly applicable to urgent primary care (UPC) or urgent-care centres (UCC). Four interrelated constructs were central to defining urgency: physiological risk/severity, linguistic or relational framing, health-service characteristics, and patient-perceived need. Service configurations varied widely (nurse-led, GP-led, hybrid, differing diagnostics and hours), and empirical comparisons of effectiveness were limited.^1^

### Why definitions matter

#### Scope of practice and recognition

Current English definitions of urgent care, which focus on activity within urgent-treatment centres and NHS 111 pathways, risk overlooking the substantial volume of urgent care delivered within routine general practice. Same-day GP surgery appointments for acute illness or deterioration of long-term conditions form a major component of urgent-care workload, yet this activity is not consistently captured in national datasets. Failure to recognise and define this contribution risks undervaluing general practice within the urgent-care system, distorting workforce planning and weakening arguments for appropriate training and resourcing.

#### Data monitoring

Reliable measurement of urgent-care activity is constrained by inconsistent definitions and limited data capture. National datasets such as the NHS Urgent and Emergency Care Dashboard collate attendances from emergency departments, NHS 111, and urgent treatment centres, but exclude the substantial volume of same-day consultations managed within general practice. The Urgent Care Data Set (UCDS) records activity by provider type rather than by clinical urgency, effectively defining ‘urgent care’ administratively.^13^ In primary care, only partial information exists: the GP Appointments Data (GPAD) collection in England reports the proportion of appointments occurring on the same day as booking, yet does not specify clinical indication, urgency, or outcome.^14^ Equivalent data are not routinely published in Scotland, Wales, or Northern Ireland, leaving much of the urgent-care workload in general practice invisible to national monitoring.

#### Workforce planning and training

As training pathways and the workforce continue to evolve,^15^ there is an increasing need for clarity in the definition of *what urgent care itself is*. Only once this has been established can consistent definitions of roles, scopes of practice, and competencies be developed. Underpinning curriculum and competency frameworks should then articulate the required skill sets and support the development of senior practitioners with sufficient expertise to provide effective ‘top cover’ for junior team members in the long term.^16^ Without such clarity, the system risks the erosion of true expertise, resulting in avoidable harm to patients, inefficiency, and rising rates of unnecessary referral.

#### Patient navigation

Inconsistent terminology creates confusion about where to seek care. Surveys consistently show uncertainty distinguishing between urgent-treatment centres, walk-in centres, and minor injury units, contributing to avoidable ED attendances and under-utilisation of community services.^17^ Variation across the UK nations further complicates access, particularly in border and tourist regions such as North Wales, where patients’ expectations often clash with local service configurations. Without a coherent national language for urgency, patients cannot reliably choose the right service at the right time.

#### System coherence

Definitional clarity is more than a semantic exercise; it is foundational to how services are commissioned, evaluated, and improved. A shared understanding of ‘urgent care’ would align data standards, enable accurate workload measurement, and support equitable resource allocation across the system. It would also give policymakers a firmer basis for evaluating reforms such as same-day access hubs and urgent primary care pathways. Without such clarity, the NHS continues to operate multiple ‘front doors’ without a common map

#### International comparison

In the US, ‘urgent care’ typically refers to standalone clinics providing walk-in care for minor conditions within extended hours.^18^ In the Netherlands and Scandinavia, the term ‘out-of-hours primary care’ denotes GP-led services rather than multidisciplinary walk-in models.^19^ This lack of definitional precision undermines international benchmarking and comparative research.

## Conceptual dimensions of urgent care

Synthesis of policy and research sources reveals four recurring domains underpinning ‘urgent care’ (Box 3).

**Box 3.** Conceptual domains underpinning the definition of urgent care

Domain	Core question	Defining features	Illustrative examples
Temporal	*How soon must care be provided?*	Time sensitivity, usually same day or within a number of hours	‘Bookable today’, ‘see within 8 hours’, ‘rapid triage required‘
Clinical severity	*What is the risk if care is delayed?*	Non-life threatening but at risk of deterioration	Exacerbation of chronic obstructive pulmonary disease, wound infection, acute pain
Service configuration	*Where and by whom is care delivered?*	Provider type, access route, and staffing model	GP-led same-day clinic, nurse-led urgent-treatment centre, NHS 111
Patient perception	*Why does the patient believe the problem is urgent?*	Subjective sense of need or risk; social and contextual drivers	Parental concern for child, anxiety after symptom escalation, limited local services

These domains interact dynamically: a condition perceived as urgent by a patient may not meet clinical thresholds, while apparently minor symptoms may conceal significant risk. Policy frameworks that define urgency by service type rather than by clinical or temporal parameters risk obscuring this nuance. Developing a consensus definition grounded in these dimensions would support coherent planning, enable meaningful comparison, and improve patient navigation across the system. The increasing use of online consultation systems and experimentation with algorithm-supported triage further highlights the need for clarity. Without a shared, clinically grounded understanding of what constitutes urgent care, such systems are likely to reproduce existing assumptions and potentially amplify regional variation through non-evidence-based tool and pathway evolution, rather than resolve it.

## Conclusion

The UK has multiple front doors for urgent problems but no shared understanding of what ‘urgent’ means. This ambiguity fragments care, distorts data, and complicates training and workforce planning. For patients, it translates into uncertainty; about where to go, whom to contact, and what response to expect. When urgency is defined by service type rather than by clinical risk or time-to-harm, both clinicians and patients lose clarity.

Addressing this requires more than semantic agreement. Leadership in developing a consensus definition should be shared between national health departments and professional bodies, with meaningful input from primary care, urgent and emergency care clinicians, service commissioners, and health services researchers. A UK-wide conceptual framework, aligned across the four nations but allowing devolved and regional flexibility in delivery, would provide a common language for policy, data collection**,** training, and service evaluation without undermining local autonomy.

A single, shared definition of *urgent care*, one grounded in clinical risk, temporal need, and patient perspective, would support safer triage, coherent workforce models, and meaningful national monitoring. It would also enable patients to make informed choices and access care in the right place, at the right time. Clear language is not a bureaucratic nicety but a prerequisite for safe, equitable, and navigable care across the NHS. While international consensus is not essential for service delivery, greater definitional clarity would strengthen international benchmarking and comparative health services research.
